# Epidemiology of Sepsis in Germany: Incidence, Mortality And Associated Costs of Care 2007-2013

**DOI:** 10.1186/2197-425X-3-S1-A50

**Published:** 2015-10-01

**Authors:** C Fleischmann, M Hartmann, CS Hartog, T Welte, S Heublein, D Thomas-Rueddel, U Dennler, K Reinhart

**Affiliations:** Jena University Hospital, Department for Anesthesiology and Intensive Care Medicine, Jena, Germany; Jena University Hospital, Center for Sepsis Control and Care (CSCC), Jena, Germany; Jena University Hospital, Hospital Pharmacy, Jena, Germany; Hannover Medical School, Clinic for Pneumology, Hannover, Germany; German Center for Lung Research, Gießen, Germany; Jena University Hospital, Geschäftsbereich Medizincontrolling, Jena, Germany

## Introduction

Sepsis is a life-threatening condition following acute infections. It is a global public health disaster. in Germany, epidemiological data on incidence, mortality and costs of sepsis is scarce.

## Objective

To assess the development of sepsis incidence, mortality and costs of care in Germany.

## Methods

We performed a nation-wide analysis of coded sepsis cases from 2007 to 2013 based on German Diagnosis Related Groups Statistics, Hospital Statistics, and Causes of Death Statistics. Sepsis cases were identified by a) clinical sepsis codes for sepsis, severe sepsis and septic shock (R65.0!, R65.1!, R57.2, respectively, as secondary diagnosis) from the DRG Statistics and b) microbiological sepsis codes (A02.1, A20.0, A20.7, A21.7, A22.7, A24.1, A26.7, A28.2, A32.7, A39.2, A39.3, A39.4, A39.1,, A40.-, A41.-, A42.7, A48.3, B00.7, A54.8, B37.7, B37.6, B49, A49.9, P36.-, O75.3, O85, R57.2 as primary diagnosis) from the Hospital Statistics and Causes of Death Statistics. Costs were estimated based on data issued by the German Federal (Social) Insurance Office.

## Results

**Figure 1:**
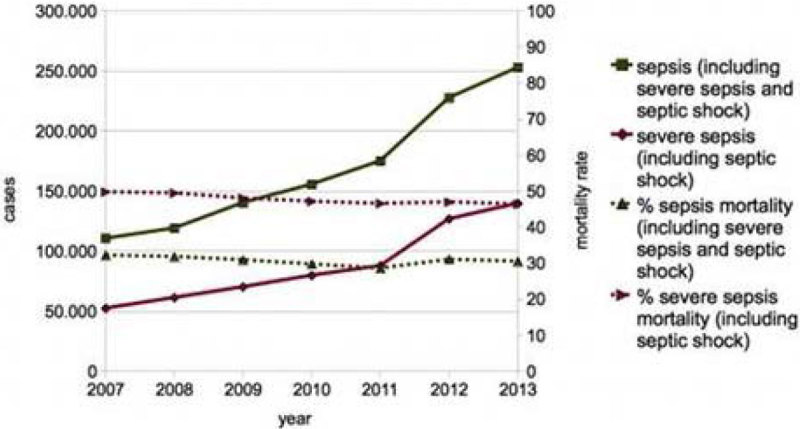
**Cases and deaths from sepsis in Germany, 20.**

Between 2007 and 2013, the incidence of sepsis increased by a mean of 15% per year from 110,653 to 252,812 cases (figure 1), yielding national estimates of 134 to 314 cases per 100.000 population. Mortality was 30.5% in 2013 and decreased on average by only 0.8% per year, resulting in over 75,000 annual deaths in 2013. Severe sepsis occurred in 55% of sepsis patients and 173 cases per 100,000 population, with a mortality of 46.4%. Hence, sepsis ranks third among the most frequent causes of death in Germany. Average direct costs per patient increased from 27,105 in 2007 to 36,129 Euros in 2012. Approximated total costs rose from 3 to 8.2 billion Euros in 2012 and can be extrapolated to 9.1 billion Euros in 2013. This is equivalent to 3% of the national health care budget. Hospital Statistics using microbiological sepsis codes considerably underestimate the incidence derived from clinical sepsis codes. The official Causes of Deaths Statistics listed merely 8.225 deaths from sepsis in 2013, thereby considerably underrepresenting the death toll from sepsis.

## Conclusions

The incidence of sepsis in Germany is higher than expected and on the rise. Demographical change and the advances in medical care and intensive care in particular may have contributed to this increase of sepsis cases, as well as better awareness and reimbursement incentives may have influenced coding practices. Mortality has not declined substantially. Transsectoral quality measures in other countries have contributed to a considerable decrease of mortality and similar measures are urgently needed in Germany. Monitoring of sepsis indices should become a regular feature of the Federal Health Monitoring and Hospital Statistics reporting.

## Grant Acknowledgment

The CSCC is funded by the German Federal Ministry of Education and Research (BMBF), Germany, FKZ: 01EO1002.

